# Pellegra

**Published:** 1915-08

**Authors:** Geo. C. Mizell

**Affiliations:** Atlanta National Bank Bldg., Atlanta, Ga.


### *PELLAGRA.

### Presenting Five Patients. Importance of Diet and Weight in Prognosis.

By Geo. C. Mizell.

Mr. President and Gentlemen of the Chattahoochee Valley Medical and Surgical Association: I wish to introduce to you

---

*Read before the Chattahoochee Valley Medical Association, July 12-13, 1915, at Opelika, Alabama.

five former patients who have been kind enough to lay aside their various duties at much inconvenience to themselves and families that they might appear before you today and acknowledge that they have been the victims of the horrible scourge of pallagra, which is now numbering its victims by the hundreds of thousands.

In their behalf and for the sakes of many others, I wish to beg you as physicians to do all you can to correct the general impression that this disease occurs only among certain classes and only in the underfed, and that those who are able to buy peas, beans and meat or milk do not have the disease. It is not unusual to hear an intelligent layman assert that should he have the disease, humiliation would force him to commit suicide. Physicians and friends of the patient are afraid to tell their pellagrins what they are suffering from. No doubt many are driven to suicide by the eroneous idea that the disease is incurable. A charlatan in this great State of Alabama is reaping a fortune by supplying a straw for the miserable victims to grasp. The medical profession, while not to blame for this state of affairs, can and should do something to correct it.

All of us who have much to do with pellagra know that some pelagrins get well, yet there are many who are skeptical. Some go so far as to say victims of pellagra cannot get well; others seeing cases recover from an exacerbation of the disease and perhaps remain free from symptoms for one or two years still believe that it will return. On the other hand, there are those who treating a case through an exacerbation conclude that the treatment has cured the disease, only to see it return in one or two years. Symptoms appear in season and disappear in season. We ask why? A patient is desperately ill with the disease a few months, recovers and goes about his business to be attacked when he is apparently well again. Another patient is not so sick, but he is continually incapaciated for work. Still another patient shows marked symptoms and is not incapacitated at all. One member of a family has the disease, another has not. Some cases have dermatitis while others have none. A healthy mother gives birth to a pellagrous child. We suspect an article of diet and exclude it, yet the patient shows annual exacerbations. Again we ask, why?

Time permitting I could add a number of questions to this riddle, and when I am through if there are any who doubt, I will be glad to be informed of the cause of their doubt.

We appear before you today so that we may add our efforts to the labors of the vast army of investigators who have been and are still searching for the truth.

The important points in the history of these patients you will find tabulated on the chart shown herewith.

Such histories as these are not unusual among my cases. These are, with one exception, patients that appeared before the Fulton County Medical Society two years ago. Case No. 1 had at the time an extensive dermatitis. Some of the members of the above mentioned medical society will doubtless recall him.

Patient No. 2 was seen with me in May, 1912, by Dr. Cosby Swanson and Dr. C. W. Gould.

Case No. 4 was seen in 1912, by Dr. R. T. Dorsey and with me by Dr. Gould.

Case No. 5 was seen in July, 1912, by Dr. M. G. Campbell, and at an earlier date by Dr. J. J. Foster.

When first seen by me, cases Nos. 2, 3, and 4, were bedridden, and No. 5 had been in bed thirty months.

You have these patients before you so that you may judge whether they are well. Their more recent histories will aid a conclusion.

Case No. 1 has been actively engaged on his farm, doing heavy work in the sun for two years. Case No. 2 has been working for two years. Case No. 3 has been attending to his business which keeps him in the sun, for three years. Case No. 4, who was incapacitated, has been doing all the housework for a family of five for nearly three years. She is never sick beyond a little headache. Case No. 5, an invalid for 2 1-2 years, has been carrying on his business as contractor for 2 1-2 years.

Are they still pellagrins? If not, what has stopped the manifestation of the disease after 2, 4 and 6 consecutive years? They drink the same water as before; they live in the same neighborhood; they are bitten by the same mosquitoes, fleas, etc. The same sun, moon and stars still shine on them; their tonsils have not been removed or treated. Yet they no longer manifest symptoms as before. What has brought about this cessation of symptoms?

Now let us grant for the present that diet is the cause of pallagra and study the following propositions:

There are two classes of pallagrins. First, those who have no constitutional symptoms. These use up all the pellagrous food and do not store up pallagrous tissue except in the skin. Second, those who have acute exacerbations. These have gained weight while taking pellagrous food. A case of one class may merge into the other. Either class may have dermatitis, but all cases of pellagra since pellagra belong to the second class. Case No. 1 of this series was of the first class for three years, then through gain in weight merged into the second class in the fourth year. Cases No. 2 and No. 3, gained weight before the first exacerbation and had acute attacks to begin with.

Let us study case No. 4, from this view point: She had her first exacerbation after taking on pallagrous material. In the attack she lost all or part of this unstable tissue. Then during a period of a few months she again took on flesh of the same material and when this process of slow saturation reached a certain point and season, she had another exacerbation. This process was repeated again and again for six years. In the last attack, which occurred in 1912, her diet was corrected and she has remained well.

I wish to explain briefly now what I mean by pellagrous tissue. When a hog or horse is fed largely on corn or cotton seed products he is not as strong an animal as when fed otherwise. He overheats easily and does not resist disease. Evidently there is something wrong with the feed. So with man; when he is fed an excess of similar food he puts on unstable flesh and his tissue becomes unstable. Such tissue does not mean disease per se, and it serves its purpose fairly well under favorable conditions, but under unfavorable conditions it permits disease. On the other hand, it appears to be so unstable that it undergoes spontaneous combustion once the functions are disturbed. The skin does not serve its purpose as a protection; it sunburns easily; it is easily irritated by pressure; by bodily secretions and by strong soaps and washing powders; also X-ray and reflected sunlight from snow. The unstable mucous tract has a tendency to break down. The intestinal mucosa permits the passage of all the toxic materials, which arise both in healthy and faulty digestion, thus giving rise to all the symptoms which are present in acidosis and gastr-intestinal auto-intoxication. In fact, there are no symptoms present in pellagra which do not also incur to some extent in gastro-intestinal auto-intoxication. Witness the statements of Combe (Pp. 161 Auto-intoxication, Rebman). "In auto-intoxication the skin becomes dry and disquamatic, the dead epithelial cells not yet replaced give the skin a dirty color," the difference here is in degree only. On p. 157, Combe states further: "In the courst of auto-intoxication we have so frequently observed probias, somber ideas, misanthrophy and even melancholia, that we are quite willing to accept its influence in psychic affections." On page 139 to 142, he describes salivary crises gastric crises, diarrheal crises and bilious crisis—such as we frequently see in pellagra. I have seen several cases in which hallucinations cleared up when indican disappeared from the urine.

The close relationship between gastro-intestinal auto-intoxication and pellagra have brought about no end of confusion. Then, too, acidosis, which is also very common in pellagra has been another source of confusion. It is becoming the fashion to make a diagnosis of "Pellagra sine Pellagra" in all cases of gastro-intestinal crises and acidosis which exhibit a red tongue whether it occurs as a post-operative condition, post partum or otherwise. Excessive carbohydrate feeding will produce symptoms which cannot be differentiated from the so-called "Pellagra sine Pellagra."

Recognize these facts and we will have a clearer vision of the problem. No mystery remains when pellagra is shown to be, first; unstable tissue; second, unstable tissue plus gastro-intestinal auto-intoxication; third, unstable tissue plus gastro-intestinal auto-intoxication and acidosis.

Only a limited amount of evidence can be offered here to show that change in the fatty material of the tissue is responsible for the unstable tissue, but evidence is abundant. Before 1914 nearly all cases of pellagra occurred in people who were otherwise unhealthy, which is equivalent to saying that it occurred in those only whose weight varied almost annually and whose skin was inactive. They were chronics who put on flesh in good times and lost it in an exacerbation of the chronic condition. Active men of even weight were long in being effected; they do not accumulate fat. Since the first of 1912 pellagra is appearing to a great extent in children. Healthy growing children in Orphanages are showing only the unstable skins. No Gastro-intestinal auto-intoxication or acidosis yet, hence no constitutional symptoms or exacerbations. These symptoms will come with further wear and tear and further saturation.

| Case No. | 1 | 2 | 3 | 4 | 5 |
|---|---|---|---|---|---|
| Age | 63 | 51 | 28 | 34 | 36 |
| Onset | 1910 | 1911 | 1911 | 1907 | 1907 |
| Duration in Years | 4 | 2 | 2 | 6 | 6 |
| No Symptoms in Mon's | 22 | 26 | 36 | 33 | 30 |
| Indicanuria | X | X | X | X | X |
| Gastric Acidity | 0 | 0 | 62 | 58 | 0 |
| Loss in Weight, Lbs. | 30 | 37 | 65 | 42 | 42 |
| Mental | — | X | — | X | X |
| Neuritis | — | X | — | — | X |

Dermatitis, Stomatitis, Esophagitis, Gastritis, Entero-Colitis, Etc.

Now, I wish again to call your attention to the loss in weight of these five cases. (See table). They have had no further exacerbations since they came under observation and the reasous are two in number; first, loss of weight; second: change in diet.

Some cases continue to have exacerbations (1, 2 or 3 years) after they come under observation. The number of years is determined by the weight lost. When they are not very sick and lose little weight the dermatitis may appear for several years even though the diet is corrected. Evidently the cause is stored up in the body and why not in the fatty deposits which may remain intact until such time as it is drawn upon to supply nourishment in acute illnesses after operations, post-partum or because of faulty digestion. The fatty tissue is the only storehouse in the animal body.

Is it not clear then from this point why in a family, one member alone is afflicted? Is it not clear why an individual may have an attack months, even years, after discontinuing the use of the unstable adipose builder? A child comes into the world with fat built from the fat of the mother's food. This fat is stable as long as it is bottled or sealed in its envelope of proteid.

Next let us consider the history of pellagra in two of our institutions. In 1911 I went out to see five cases of pellagra at the Methodist Orphanage located at Decatur, Ga. (Two cases had a recurrence in 1912 and one in 1913). The Superintendent informed me that Dr. H. F. Harris, who had preceded me, found 15 cases. There has not been a case of pellagra in two years and no new cases in four years.

Dr. Geo. C. Trimble, Physician to the Georgia Baptist Orphanage at Hapeville, Ga., gives the history of pellagra as follows: (Atlanta Journal Record of Medicine Vol. LXI, No. 9.) "In 1911—22 cases. In 1913—75 cases. In 1915—4 recurrences, 2 new cases. No accurate figures are given by Dr. Trimble in his paper as to residence of the children in the institution, and no complete figures of recurrence or new cases, but from personal investigation I can say that there are new cases and recurrences each year with a diminished number so far this year. Dr. Trimble states that the children have had a wholesome, varied diet but the present Superintendent told me that it had been much better since September, 1914.

The points are, first: that pellagra began in both institutions in 1911; second, the management in the Decatur Orphanage corrected the diet at once and pellagra disappeared two years ago, just as in those patients exhibited here today. In Hapeville Orphanage the diet was not corrected and pellagra persists. We could give you hundreds of individual instances. And, furthermore, no new cases of pellagra have developed in the families of these hundreds of individual cases.

In conclusion let me state this; first, that the association of corn with ancient pellagra is no myth; second, that the distinctive constituent of corn is the fat or oil which differs only in the proportion of the fatty acids from the other oils or fats of the group to which it belongs. Third, all pellagrins eat excessively for a long time of some of these fats. Fourth, when the consumption of these is limited or excluded, pellagra disappears in due time.

Atlanta National Bank Bldg., Atlanta, Ga.

